# A Fluorescence Inner-Filter Effect Based Sensing Platform for Turn-On Detection of Glutathione in Human Serum

**DOI:** 10.3390/s19020228

**Published:** 2019-01-09

**Authors:** Shurong Tang, Xiuhua You, Quanhui Fang, Xin Li, Guangwen Li, Jinghua Chen, Wei Chen

**Affiliations:** Department of Pharmaceutical Analysis, Faculty of Pharmacy, Fujian Medical University, Fuzhou 350108, China; hhyou4906@163.com (X.Y.); 18250485385@163.com (Q.F.); lx281032@163.com (X.L.); lgw6301@fjmu.edu.cn (G.L.); cjh_huaxue@126.com (J.C.); chenandhu@163.com (W.C.)

**Keywords:** fluorescence inner-filter effect, manganese dioxide nanosheets, molybdenum disulfide quantum dots, glutathione detection, human serum

## Abstract

A novel turn-on fluorescence assay was developed for the rapid detection of glutathione (GSH) based on the inner-filter effect (IFE) and redox reaction. Molybdenum disulfide quantum dots (MoS_2_ QDs), which have stable fluorescent properties, were synthesized with hydrothermal method. Manganese dioxide nanosheets (MnO_2_ NSs) were prepared by exfoliating the bulk δ-MnO_2_ material in bovine serum albumin (BSA) aqueous solution. The morphology structures of the prepared nanoparticles were characterized by transmission electron microscope (TEM). Studies have shown that the fluorescence of MoS_2_ QDs could be quenched in the presence of MnO_2_ NSs as a result of the IFE, and is recovered after the addition of GSH to dissolve the MnO_2_ NSs. The fluorescence intensity showed a good linear relationship with the GSH concentration in the range 20–2500 μM, the limit of detection was 1.0 μM. The detection method was applied to the analysis of GSH in human serum samples. This simple, rapid, and cost-effective method has great potential in analyzing GSH and in disease diagnosis.

## 1. Introduction

Glutathione (GSH), the most abundant intracellular low-molecular-weight biothiol, is a tripeptide consisting of cysteine, glutamic acid, and glycine. As an essential endogenous antioxidant, GSH plays crucial roles in biological systems and many cellular processes, such as maintaining the appropriate intracellular redox status, protecting against many toxins, and promoting metabolism [1]. Abnormal GSH levels in the blood are closely related with some clinical diseases including leucocyte loss, inflammation, liver damage, psoriasis, Parkinson’s, Alzheimer’s, and cancer [2,3]. It has been found that the GSH level is significantly elevated in cancer cells [4]. Therefore, developing a simple and sensitive method for GSH detection in human serum is critically important for understanding GSH homeostasis in clinical settings and in effective cancer diagnosis at an early stage.

To date, a number of analytical methods have been established for GSH measurement, including high-performance liquid chromatography [5], mass spectrometry [6], surface enhanced Raman scattering [7], enzyme-linked immunosorbent assay [8], electrochemical analysis [9], and fluorescence spectroscopy [10]. Fluorescence spectrometry has been found to be a powerful tool for GSH detection because of its simplicity, high sensitivity, good flexibility, and nondestructive readout [11,12]. Several fluorescence assays have been developed for estimating GSH levels in biological samples [13,14]. Most of these assays are based on fluorescence “turn-off” patterns or organic dye probes, which make them susceptible to false positive results, lowered sensitivity, or poor photostability. Thus, we still need to establish a novel fluorescence “turn-on” method for the sensing of GSH.

Owing to the exceptional advantages, fluorescent nanomaterials have recently gained great attention as promising substitutes for organic dyes, such as quantum dots [15,16], gold nanoclusters [17,18], carbon dots [19,20], and upconversion nanoparticles [21]. Among them, quantum dots (QDs), which display superior properties including a high-fluorescence quantum yield, size/composition-dependent tunable luminescence, broad excitation/narrow emission spectrum, excellent stability, and good resistance to photobleaching [22], have become ideal probes for signal transduction in the fluorescent assays. For instance, CdSe@ZnS QDs have been used to construct a fluorescence lateral flow biosensor for the rapid detection of GSH [16]. Crystal violet-functionalized CdTe QDs were synthesized for detecting GSH in water [23]. Au nanocluster based fluorescent sensor has been designed for turn-on sensing of GSH, in which Hg^2+^ acted as a quencher [24]. The introduction of highly toxic heavy metal ions (Cd^2+^ or Hg^2+^) into these systems posed a big threat to the environment. MoS_2_ QDs, novel fluorescent nanoparticles with ideal environmental compatibility, high fluorescent intensity, easy operability, and a low cost are a promising candidate for GSH analysis [25,26,27].

Conventional sensing systems based on fluorescence resonance energy transfer (FRET) have been used to detect various analytes. However, they only work when the fluorophore and quencher are at a particular distance, which restrict the application of FRET-based fluorescence assays. The fluorescence inner-filter effect (IFE) refers to the absorption of the excitation/emission light of fluorophores by appropriate absorbers [28]. The sensitivity of the IFE system can be enhanced due to that the fluorescence of fluorophore would be exponentially changed with the absorbance of absorber [29]. MnO_2_ nanosheets (NSs), a new class of two-dimensional nanomaterials, have received increasing attention due to their attractive characteristics, including their high specific surface area, broad absorption spectrum (200–600 nm), large extinction coefficient (9.6 × 10^3^ M^−1^ cm^−1^) [30], unique redox activity, stability, and biocompatibility [31]. These features enable them to be used as a potentially ideal fluorescent absorber in the IFE-based assay.

Herein, we developed a simple “turn-on” fluorescence assay for the rapid detection of GSH based on the IFE. In this system, MoS_2_ QDs were used as the fluorophore and MnO_2_ NSs as the absorber. The existence of GSH can cause the decomposition of MnO_2_ NSs, leading to a concentration-dependent reduction of MnO_2_ and proportional increase of the fluorescence. This assay can be conveniently used for the sensing of GSH in human blood serum, which holds great promise for biomedical applications.

## 2. Materials and Methods

### 2.1. Materials and Instruments

Hydrogen peroxide (H_2_O_2_) was purchased from Hengxing Chemical Reagent Co., Ltd. (Tianjin, China). Sodium molybdate dihydrate (Na_2_MoO_4_·2H_2_O) was purchased from Phygene Biotechnology Co., Ltd. (Fuzhou, China). Manganese sulfate monohydrate (MnSO_4_·H_2_O), magnesium chloride hexahydrate (MgCl_2_·6H_2_O), sodium chloride (NaCl), calcium chloride (CaCl_2_), copper sulfate (CuSO_4_), and hydrochloric acid (HCl) were purchased from the Sinopharm Chemical Reagent Co., Ltd. (Shanghai, China). Bovine serum albumin (BSA), tetramethylammonium hydroxide (TMA·OH), glutathione (GSH), oxidized glutathione (GSSG), l-glutamate (l-Glu), l-histidine (l-His), l-phenylalanine (l-Phe), glycine (Gly), cystine (3,3′-Dith), l-methionine (l-Met), and dl-α-aminopropionic acid (dl-α-Ala) were purchased from Aladdin Reagent Company (Shanghai, China). The reagents used were all analytical grade. Human serum was kindly provided by the Hospital of Fujian Medical University. Deionized water was prepared by Milli-Q Water Purification System for all the experiments.

The absorption spectra were conducted on a UV-2600 UV-vis spectrophotometer (Shimadzu, Tokyo, Japan). Fluorescence spectrum was recorded on an F-4600 fluorescence spectrophotometer (Hitachi, Tokyo, Japan). High-resolution transmission electron microscopic (HRTEM) imaging was performed on a JEOL 2100F field emission transmission electron microscope (JEOL, Tokyo, Japan). The dynamic light scattering (DLS) experiment was performed on a Zetasizer Nano-ZS90 (Malvern, Worcestershire, UK). The XRD pattern was obtained by a D8 Advance X-ray diffractometer (Bruker, Karlsruhe, Germany). Absolute fluorescence quantum yields (QY) measurements were conducted on an Edinburgh FLS 980 spectrometer with an integrating sphere (Edinburgh, Livingston, UK).

### 2.2. Preparation of Bulk δ-MnO_2_ and MnO_2_ NSs

Bulk δ-MnO_2_ was prepared as reported previously [32]. Typically, 20 mL of a mixture containing TMA·OH (0.6 M) and H_2_O_2_ (3 wt %) was added into 10 mL of MnSO_4_ (0.3 M) aqueous solution within 15 s. The as-prepared dark brown solution was stirred vigorously overnight at room temperature. Then, the resulting precipitate was removed by centrifugation at 10,000 rpm for 10 min, and washed three times with ultrapure water. Finally, the precipitate was dried in a vacuum oven at 30 °C.

The MnO_2_ NSs were prepared by exfoliating the bulk δ-MnO_2_ in BSA aqueous solution [33]. Briefly, 10 mg bulk δ-MnO_2_ was added into a 10 mL BSA (1 mg/mL) aqueous solution and the mixture was sonicated for 12 h. Then, the suspension was centrifuged at 1000 rpm for 20 min to remove the unexfoliated MnO_2_. The supernatant, which contained the MnO_2_ NSs, was collected and stored in a refrigerator at 4 °C.

### 2.3. Preparation of MoS_2_ QDs

MoS_2_ QDs were synthesized by a hydrothermal method according to the literature [34] with some modifications. Concisely, 0.25 g of Na_2_MoO_4_·2H_2_O was dissolved in 25 mL of water and subjected to ultrasonication for 5 min. Then, the solution was adjusted to pH 6.5 with 0.1 M HCl. After that, 0.5 g Cys and 50 mL water were added into the solution followed by ultrasonication for 10 min. The mixture was then heated in a Teflon-lined stainless steel autoclave at 200 °C for 36 h. After cooling to room temperature, the supernatant was collected after being centrifuged at 12,000 rpm for 30 min. Finally, the stable MoS_2_ QDs were obtained after dialyzed for 19 h to remove the impurity using dialysis bag (3500 Da).

### 2.4. Detection of GSH

Firstly, 5 μL of GSH aqueous solution with different concentrations was added into 100 μL of 0.2 M acetate buffer (pH = 5.0) in a low adsorption tube. Then, 15 μL of MnO_2_ NSs (1.0 mM) was added and the mixture was shaken and reacted for 10 min. Next, 80 μL of MoS_2_ QDs (1.6 mg/mL) was added into the as-prepared solution, shaking well to ensure complete reaction. Finally, the fluorescence intensity of the whole reaction solution was detected at room temperature (Ex: 320 nm, Em: 340–600 nm, the peak was about 400 nm). The specific experiments were conducted in the same way, except using other amino acid instead of GSH solution.

### 2.5. Analysis of GSH in Human Serum Samples

The standard addition method was used to analyze the GSH level in human serum. Briefly, 95 μL of 0.2 M acetate buffer (pH = 5.0), 5 μL different concentrations of GSH aqueous solution, 5 μL human serum, and 15 μL of MnO_2_ NSs were added into a low-adsorption tube. After being reacted for 10 min, 80 μL of MoS_2_ QDs was added into the above mixture, shaking well to ensure complete reaction. Finally, the fluorescence intensity of the resulting solution was measured. The whole process was carried out at room temperature.

## 3. Results and Discussion

### 3.1. Fluorescence Sensing Principle of GSH

**Scheme 1:**
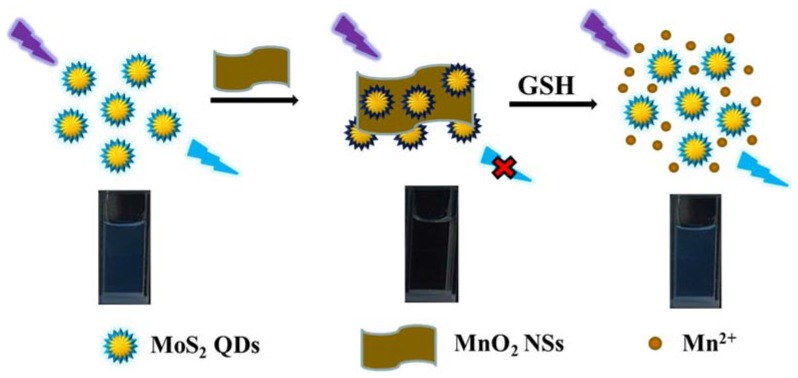
Schematic diagram of the fluorescence assay for GSH detection.

The sensing principle of GSH is shown in Scheme 1. The synthesized MoS_2_ QDs emitted blue fluorescence under irradiation at 320 nm. MnO_2_ NSs possessed a broad absorption spectrum and large molar extinction coefficient, making them a promising quencher in the IFE. After the addition of MnO_2_ NSs, the fluorescence of MoS_2_ QDs was effectively quenched as a result of the IFE. In the presence of reductive GSH, the MnO_2_ NSs (strong oxidation ability) can be decomposed through the redox reaction. The released Mn^2+^ lost its quenching ability, thus the fluorescence of MoS_2_ QDs was recovered. On the basis of this mechanism, a “turn-on” fluorescent detection method was developed for the rapid sensing of GSH in human serum.

### 3.2. Characterization of MoS_2_ QDs and MnO_2_ NSs

**Figure 1:**
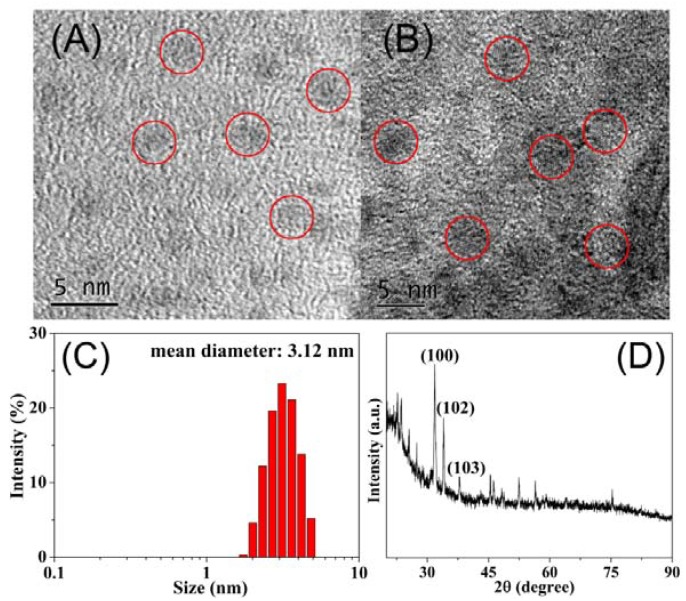
(**A**) TEM; (**B**) high-resolution transmission electron microscopic (HRTEM) image; (**C**) dynamic light scattering (DLS) analysis; and (**D**) XRD pattern of MoS_2_ quantum dots (QDs).

**Figure 2:**
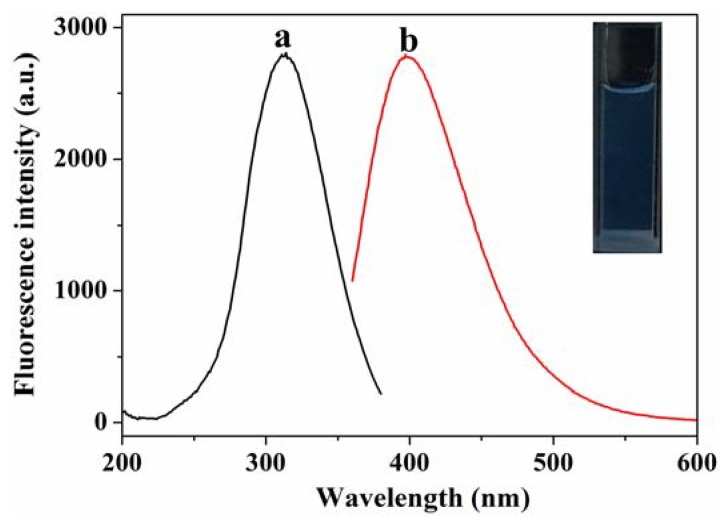
Fluorescence excitation (**a**) and emission (**b**) spectra of MoS_2_ QDs (Inset: The photo of MoS_2_ QDs solution under UV irradiation). Detection parameters: Ex slit, 5.0 nm; Em slit, 10.0 nm; Scan speed, 1200 nm/min; PMT, 700 V.

**Figure 3:**
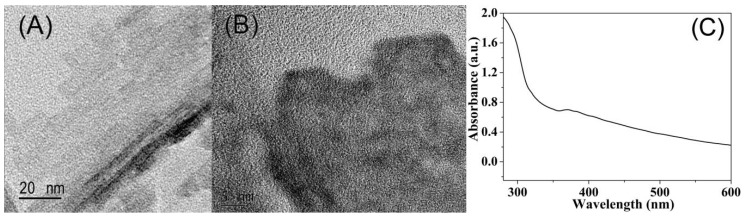
(**A**) TEM; (**B**) HRTEM image; and (**C**) UV-Vis spectrum of manganese dioxide nanosheets (MnO_2_ NSs).

The morphologies of MoS_2_ QDs and MnO_2_ NSs were characterized by transmission electron microscopy (TEM). TEM (Figure 1A) and DLS (Figure 1C) analysis results show that the synthesized MoS_2_ QDs are well dispersed with the particle size of about 3 nm. The HRTEM image shows that the MoS_2_ QDs has obvious lattice fringes (Figure 1B). Figure 1D is the XRD spectrum of MoS_2_ QDs. Three major diffraction peaks attributed to (100), (102), and (103) planes of MoS_2_ can be observed [34]. The fluorescence properties of the MoS_2_ QDs were further investigated. From Figure 2, it was clearly observed that the most intense peak of MoS_2_ QDs appeared at 320 nm in the excitation spectrum (curve a). Under this excitation light, a well distinguishable peak with maximum emission intensity at 400 nm appeared (curve b). The MoS_2_ QDs solution emitted blue fluorescence when exposure to a 365 nm UV lamp (inset Figure 2). As shown in Figure 3A, an ultrathin lamellar structure with an irregular edge of MnO_2_ NSs was observed. Figure 3B shows the HRTEM image of MnO_2_ NSs with the diffraction pattern. The UV-Vis spectrum shows that the prepared MnO_2_ NSs possessed a broad absorption in the range 200–600 nm (Figure 3C).

### 3.3. Feasibility Analysis of GSH Detection

**Figure 4:**
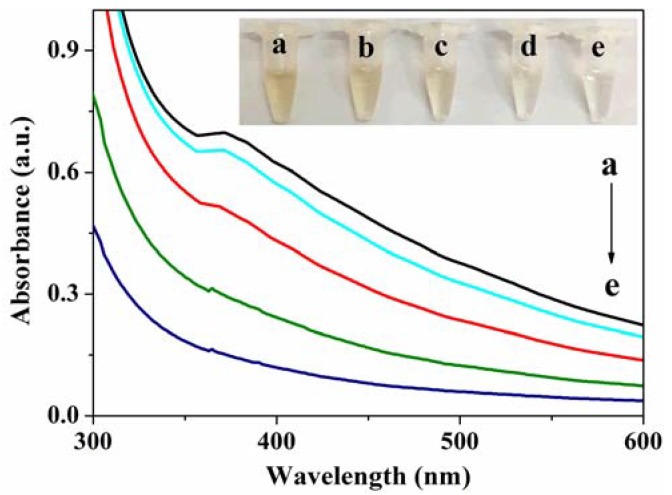
UV-Vis spectra of MnO_2_ NSs solution (0.075 mM) after the addition of different concentrations of GSH (a: 0, b: 100, c: 500, d: 2000, e: 5000 µM) (Inset: The corresponding photographs of the above solutions).

To investigate the ability of GSH to dissolve MnO_2_ NSs, different concentrations of GSH were added into the MnO_2_ NSs, and then the solutions were analyzed by UV-Vis spectrophotometer. As shown in Figure 4, the MnO_2_ NSs has a characteristic absorption peak at about 380 nm (curve a), which is consistent with the literature [35]. With the increase of GSH concentration, the absorption intensity decreased (curve b–e) and the color of the solution became gradually shallower (inset Figure 4). These results indicated that the MnO_2_ NSs can be decomposed by GSH through the redox reaction [36]. According to the Beer-Lambert law (A = kbc, k = 9.6 × 10^3^ M^−1^ cm^−1^, b = 1 cm), the concentration of the synthetic MnO_2_ NSs was calculated to be about 1 mM.

**Figure 5:**
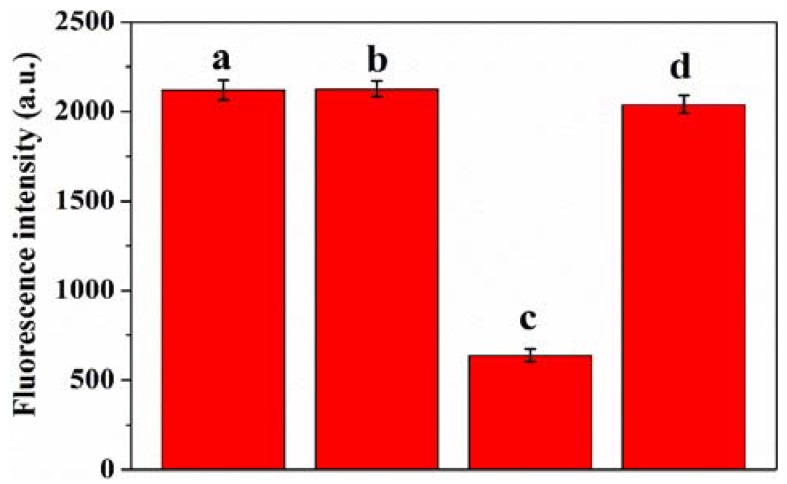
The effect of MnO_2_ NSs and glutathione (GSH) on the fluorescence intensity of MoS_2_ QDs. (bar graph a: MoS_2_ QDs (0.64 mg/mL), bar graph b: MoS_2_ QDs + 5 μL 400 mM GSH, bar graph c: MoS_2_ QDs + 15 μL 1.0 mM MnO_2_ NSs, bar graph d: MoS_2_ QDs + 15 μL 1.0 mM MnO_2_ NSs + 5 μL 400 mM GSH).

In order to verify the fluorescence sensing feasibility of GSH based on the probe of MoS_2_ QDs and MnO_2_ NSs, serials experiments were performed (Figure 5). It can be seen that MoS_2_ QDs emitted strong fluorescence (bar graph a). The added GSH did not greatly change the fluorescence intensity of MoS_2_ QDs (bar graph b). After addition of MnO_2_ NSs, the fluorescence of MoS_2_ QDs was significantly quenched (bar graph c). In the presence of GSH, the MnO_2_ NSs was dissolved and thus recovered the fluorescence (bar graph d). Therefore, it can be proved that the proposed fluorescence assay was feasible for the “turn-on” detection of GSH. The QY of the MoS_2_ QDs probe and for the restored fluorescence in the presence of GSH were measured to be 2.13% and 2.39% by the absolute quantum yield measurement method, respectively.

### 3.4. Optimization of Experimental Conditions

**Figure 6:**
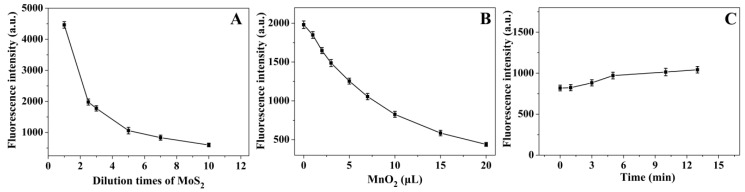
(**A**) The effect of MoS_2_ QDs dilution time (initial concentration 1.6 mg/mL); (**B**) MnO_2_ NSs concentration (1.0 mM); (**C**) and the reaction time between GSH and MnO_2_ NSs on the fluorescence intensity.

The concentration of MoS_2_ QDs and MnO_2_ NSs, used as the fluorescence and quencher probe, respectively, have a great effect on the detection sensitivity. The fluorescence intensities of different concentrations of MoS_2_ QDs solutions were determined. As shown in Figure 6A, the fluorescence intensity decreased as the dilution multiple of the MoS_2_ QDs increased, then the decreasing trend gradually tended to be gentle. Considering the sensitivity and fluorescence intensity, the final concentration of 2.5-fold diluted MoS_2_ QDs solution was used in the following experiments. As the amount of MnO_2_ NSs increased, the fluorescence of MoS_2_ QDs sharply decreased (Figure 6B). Excessive MnO_2_ NSs reduces the GSH detection sensitivity. After a comprehensive analysis of the fluorescence inhibition degree and sensitivity, 15 µL of MnO_2_ NSs was finally selected. The reaction time between GSH and MnO_2_ NSs was also investigated. The fluorescence intensity increased gradually with the time prolonging from 0 to 10 min, then it tended to be stable (Figure 6C). Therefore, the reaction time was selected to be 10 min.

### 3.5. GSH Detection Sensitivity

**Figure 7:**
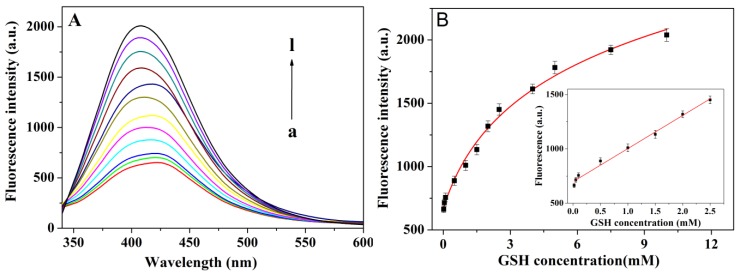
(**A**) Fluorescence spectra of the system at different GSH concentrations (From a to l: 0.02, 0.05, 0.1, 0.5, 1, 1.5, 2, 2.5, 4, 5, 7.5, and 10 mM; MoS_2_ QDs 0.64 mg/mL, MnO_2_ NSs 0.075 mM); (**B**) the relationship between GSH concentration and the fluorescence intensity (Inset: The calibration plot of fluorescence intensity against the concentrations of GSH).

To investigate the capability of the fluorescence assay for quantitative measurement of GSH, different concentrations of GSH were added to the system and the fluorescence responses were recorded under optimal conditions. The fluorescence of MoS_2_ QDs was found to be gradually restored with an increasing concentration of GSH (Figure 7A). The more GSH added, the more MnO_2_ NSs decomposition, which resulted in a restoration of the fluorescence. The standard curve was obtained between the fluorescence intensity and the GSH concentration (Figure 7B). The inset in Figure 7B shows that the fluorescence intensity was linearly related to the GSH concentration in a range from 0.02 to 2.5 mM with a correlation coefficient of 0.996. The linear equation is Y = 301.94X + 702.72, where Y is the fluorescence intensity and X is the concentration of GSH (mM). The detection limit was calculated to be 1.0 μM according to the three-fold standard deviation of the blank signal (3σ). As shown in Table 1, the detection sensitivity was comparable to that of some reported methods and could meet the requirement of GSH detection in biological and clinical applications (mM levels) [7].

### 3.6. Interference Detection Results

**Figure 8:**
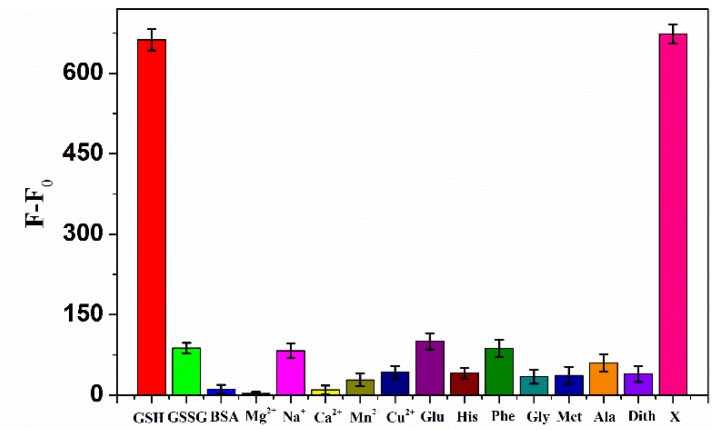
Selectivity investigation of the system for GSH detection. The concentration of GSH, oxidized glutathione (GSSG), and other amino acid solutions were 2 mM, bovine serum albumin (BSA) was 1 mg/mL, and the metal ions concentration was 5 mM. Bar graph “X” was the fluorescent recovery of the mixture of several amino acids (Glu, His, Phe, Gly, Met, Ala, and Dith with a concentration of 2 mM) with 2 mM GSH.

To assess the selectivity of GSH detection based on the MoS_2_ QDs-MnO_2_ NSs system, the influence of some amino acids, metal ions, and proteins which possibly exist in human serum was studied. As shown in Figure 8, the system exhibited a remarkable increase in fluorescence in the presence of GSH. On the contrary, there were no obvious changes in fluorescence intensity with the other molecules. It was also found that when GSH was mixed with some other amino acids, the fluorescent intensity was similar to GSH alone. Although a high concentration of cysteine (Cys) and homo-cysteine (H-Cys) can cause a measurable fluorescence response to this system, their contents (μM levels) are much lower than that of GSH (mM levels) in biological systems [41,42]. These results show that the system displays a highly selective response of fluorescence enhancement towards GSH over other molecules.

### 3.7. GSH Detection in Human Serum

Human serum experiments were further performed to investigate the feasibility of the MoS_2_ QDs-MnO_2_ NSs system in the analysis of GSH in actual samples. Different concentrations of standard GSH were spiked in the human serum and the samples were measured in parallel three times. As shown in Table 2, the recovery rate of the method was found to be 99.2–104%, and the RSD was within 10%. It is shown that this method can be used for the detection of GSH in human serum with acceptable reproducibility and accuracy.

### 3.8. Reaction Mechanism Discussion

**Figure 9:**
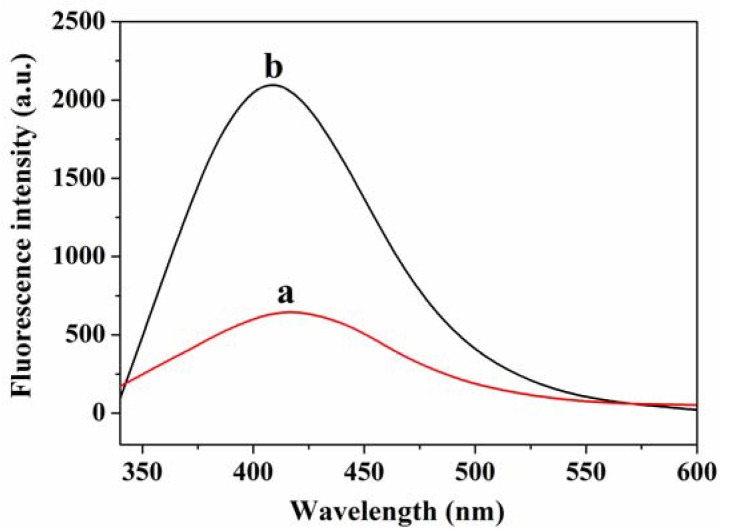
Fluorescence spectra of the solution containing MoS_2_ QDs and MnO_2_ NSs before (**a**) or after (**b**) centrifuge treatment.

The fluorescence internal filter effect refers to the phenomenon that occurs when the fluorophor coexists with other absorbents, and the fluorescence is weakened due to the absorption of the excitation or emission light by the absorbent. The broad absorption of MnO_2_ NSs possesses a peak at about 380 nm (Figure 3C), which overlaps both with the emission and excitation wavelength of MoS_2_ QDs. Therefore, when both of them exist in the system, the fluorescence of MoS_2_ QDs will be quenched due to the IFE (Figure 9, curve a). Interestingly, we found that after high-speed centrifugation of the reaction solution, the fluorescence of the supernatant was restored (Figure 9, curve b), possibly as a result of the suspended MnO_2_ NSs in the solution being separated from the MoS_2_ QDs and precipitated in the bottom of the centrifuge tube. Thus, the fluorescence of MoS_2_ QDs in the supernatant cannot be quenched by MnO_2_ NSs through the IFE. The experimental results confirmed that the detection of GSH was based on the IFE, which is different from the reported FRET mechanism of single-layer MnO_2_ nanosheets [36,43].

### 3.9. Stability Investigation

The stability of the prepared MoS_2_ QDs with and without dialysis was investigated. We found that after being placed at room temperature for 42 days, the synthesized MoS_2_ QDs after dialysis retained about 96% of their original fluorescence intensity. However, the fluorescence of MoS_2_ QDs decreased sharply without dialysis, being only 61% of the previous measurement. It shows that the stability of MoS_2_ QDs is greatly improved by dialysis treatment, which is a benefit for practical applications.

## 4. Conclusions

In summary, a signal enhanced fluorescence assay was developed for the rapid detection of GSH in human serum. The fluorescence of MoS_2_ QDs can be effectively quenched by MnO_2_ NSs as a result of the IFE. Interestingly, the quenched fluorescence was restored when reductive GSH was introduced to dissolve MnO_2_ NSs through the redox reaction. The final fluorescence intensity was linearly correlated with the concentration of GSH. The whole detection process can be completed within 15 min. Notably, the fluorescence response was selective toward GSH without interference from other substances in human serum. Thus, the assay was successfully used for the analysis of GSH in human serum samples. We anticipate that this simple, stable, selective, and cost-effective “turn-on” fluorescent assay has a good prospect of application for GSH analysis in biomedical fields.

## Figures and Tables

**Table 1:** Comparison of our present work with other methods for GSH detection.

Methods	Probes	Linear Range	LOD	References
Fluorescence	MnO_2_-UCNPs	-	0.9 μM	[36]
Fluorescence	CdSe@ZnS QDs-MnO_2_	0.05~1.5 mM	16.3 μM	[16]
Fluorescence	CDs-MnO_2_ NPs	1~200 μM	0.6 μM	[37]
Fluorescence	GQDs–MnO_2_ composite	1~1000 μM	0.45 μM	[38]
Colorimetric	MnO_2_NSs-TMB	1~25 μM	0.3 μM	[31]
Colorimetric	GSH-AuNCs-TMB	2~25 μM	0.42 μM	[39]
Electrochemistry	CdTe QDs	24~214 μM	8.3 μM	[40]
Fluorescence	MoS_2_ QDs-MnO_2_ NSs	20~2500 μM	1.0 μM	This work

**Table 2:** Analysis of GSH in human serum samples (n = 3).

Sample	Add/mM	Found/mM	Recovery/%	RSD/%
1	0	0.193	-	9.6
2	0.1	0.104	104	2.1
3	1.0	0.992	99.2	2.6
